# Adverse event profiles of adjuvant treatment with opicapone in Parkinson’s disease: A systematic review and meta-analysis

**DOI:** 10.3389/fphar.2022.1042992

**Published:** 2022-11-24

**Authors:** Luwen Xie, Xiaoyi Qi, Xuan Wang, Bing He, Yu Wang, Wei Zhang, Zehui Yu, Mingming Deng, Sicheng Liang, Muhan Lü

**Affiliations:** ^1^ Department of Gastroenterology, The Affiliated Hospital of Southwest Medical University, Luzhou, China; ^2^ Department of Dermatology, The Affiliated Hospital of Southwest Medical University, Luzhou, China; ^3^ The Public Platform of Advanced Detecting Instruments, Public Center of Experimental Technology, Southwest Medical University, Luzhou, China; ^4^ Department of Orthopedics, Gulin County People’s Hospital, Luzhou, China; ^5^ Human Microecology and Precision Diagnosis and Treatment of Luzhou Key Laboratory, Luzhou, China; ^6^ Cardiovascular and Metabolic Diseases of Sichuan Key Laboratory, Luzhou, China

**Keywords:** opicapone, Parkinson’s disease, adverse events, adjunctive treatment, meta-analysis

## Abstract

**Background:** Opicapone, a novel third-generation catechol-O-methyltransferase inhibitor, has demonstrated efficacy in Parkinson’s Disease (PD) patients with end-of-dose motor fluctuations.

**Objective:** This study aimed to compare the short-term (<6 months) and long-term (≥6 months) tolerability of opicapone adjuvant treatment in PD patients.

**Method:** Electronic databases including PubMed, Embase, Web of Science and Cochrane library were searched for randomized controlled trials (RCTs) and observational studies. The end points included any treatment-related adverse events (TEAEs), serious TEAEs (SAEs) and treatment discontinuation. A random-effects model was used to generate overall incidences of TEAE.

**Results:** Three RCTs, three RCT extension studies and three open-label studies involving 2177 PD patients were evaluated. In the short-term studies, there were reports of TEAEs with an incidence of ≥5% in individuals treated with opicapone 50 mg, including dyskinesia (14.1%), elevated blood creatine phosphokinase levels (8.0%) and urinary tract infection (6.0%). Any TEAEs, SAEs and treatment discontinuation all occurred at rates of 62.9%, 4.8% and 9.3%, respectively. TEAEs with opicapone 50 mg that were reported by more than 5% of patients in long-term studies included dyskinesia (16.1%), dry mouth (12.1%), medication effect decreased (12.1%), PD exacerbated (7.8%), blood creatine phosphokinase level raised (7.4%), nausea (6.1%) and insomnia (5.1%). The incidence of any TEAEs, SAEs and treatment discontinuation were, correspondingly, 73.2%, 8.7% and 8.4%.

**Conclusion:** These studies demonstrated that opicapone was generally well-tolerated and had a low risk of adverse events, suggesting that it could be a valuable therapeutic choice for people with PD.

## 1 Introduction

Parkinson’s disease (PD) is a progressive neurodegenerative disorder characterized by the loss of nigrostriatal dopamine and the buildup of a-synuclein (Bohnen et al., 2022; DeMaagd and Philip, 2015; Xu and Pu, 2016; O'Keeffe and Sullivan, 2018). In addition to other non-motor symptoms, typical clinical signs of PD include bradykinesia, resting tremor, muscular rigidity and postural instability (Müller, 2015; Gómez-Benito et al., 2020). PD has a significant effect on society. Approximately 6.1 million people suffer from PD in 2016 alone (GBD 2016 Neurology Collaborators, 2019; Bloem et al., 2021). In the last 2 decades, the global burden of PD has more than doubled in terms of deaths and disability (Deuschl et al., 2020). Even though dopaminergic medications continue to be the gold standard for the symptomatic therapy of PD (Hornykiewicz, 2010), there are still various unmet needs in the treatment of dopaminergic-resistant motor and non-motor symptoms, as well as therapies that alter the disease’s natural clinical course.

In addition, long-term treatment with levodopa is frequently associated with fluctuations in motor response and the development of involuntary movements such as dyskinesias (Müller, 2015). To address these issues, additional medications are needed, such as dopamine agonists, monoamine-oxidase-B inhibitors and catechol-O-methyltransferase (COMT) inhibitors (Stocchi et al., 2008).

Opicapone, a novel third-generation COMT inhibitor, has been used as adjunctive therapy in levodopa-treated PD patients (Fabbri et al., 2018). Opicapone is a purely peripheral inhibitor of COMT with an unprecedented duration of action. In addition, opicapone exhibits favorable pharmacokinetics when administrated with levodopa, resulting in higher plasma levodopa levels over prolonged periods (Kiss et al., 2010). A single daily dosage of 50 mg opicapone may be effective in the clinical setting of PD (Jenner et al., 2021). Previous COMT inhibitors, such as tolcapone, have restricted applications since liver toxicity must be considered, whereas entacapone must be taken frequently due to its short half-life (Kaakkola and Nissinen, 2010; Fabbri et al., 2016). Opicapone is aimed at solving these problems and offering an alternative way to treat end-of-dose motor fluctuations.

On 24 April 2020, the United States Food and Drug Administration approved of opicapone as an adjunctive treatment to carbidopa/levodopa therapy in patients with PD experiencing OFF episodes. However, the existing paucity of data on opicapone tolerability over the long-term phase may limit its application. The purpose of this study was to undertake a meta-analysis of the current literature to estimate the tolerability of opicapone treatment in patients with PD during the short- and long-term phases, as well as to evaluate the value of opicapone more comprehensively.

## 2 Methods and materials

The Preferred Reporting Items for Systematic Reviews and Meta-Analyses (PRISMA) statement was followed when doing this research (Page et al., 2021). The protocol for the review is available on PROSPERO (CRD42022331625) (http://www.crd.york.ac.uk/PROSPERO).

### 2.1 Search strategy

Two reviewers (XLW and QXY) independently searched PubMed, Embase, Web of Science and Cochrane library for articles by entering “Parkinson’s Disease”, “opicapone” and “Drug-Related Side Effects and Adverse Reactions.” The language was restricted to English. The search strategy is shown in Supplementary Materials. The bibliographic databases were searched from their respective inceptions to 7 June 2022. We also searched through all of the identified literature to look for more relevant studies, such as those in reference lists.

### 2.2 Study selection

The articles were included in the meta-analysis if they met the following criteria: (i) patients diagnosed with PD; (ii) PD patients treated with opicapone (50 mg); and (iii) short-term (<6 months) or long-term (≥6 months) safety was investigated. The following items were on the list of excluded criteria: (i) studies with insufficient data; (ii) conference reports, system evaluations or summary articles; and (iii) non-English literature. Disagreement between the two reviewers was settled by consensus or consultation with a third author (LSC).

### 2.3 Data extraction

The full text and extra appendices of the studies included were read independently by the two researchers (XLW and QXY). Study type, participant characteristics, sample size, dosage and adverse events were extracted independently from these studies, with differences resolved by discussion. If critical data was missing, we emailed the authors for more information. Any undesirable medical occurrence in a patient who has been given a medicinal substance, including occurrences that are not necessarily related to or caused by that medication, is referred to as an “adverse effect".

### 2.4 Assessment of the risk of bias

Two independent researchers used the Cochrane Collaboration’s Risk of Bias tool to assess the risk of bias in each trial including a six-item scale (selection, performance, detection, attrition, reporting and other biases) (Higgins et al., 2011). Each item required a judgment of high, low or unclear bias risk, with lower bias indicating higher quality. The Cochrane Handbook contains detailed guidelines for reaching judgments regarding the possibility of bias from each of the tool’s elements (Higgins et al., 2019). Any disagreement between the two reviewers was resolved by consensus or consultation with a third reviewer (LSC).

### 2.5 Statistical analysis

The metaprop command (meta-analysis of single proportions) was used to calculate treatment-related adverse events (TEAEs) rates and confidence intervals (CIs) through the random-effects model. The Chi-square test was used to measure heterogeneity; I^2^ values of less than 50% indicate no significant heterogeneity and are acceptable. Due to the small number of included papers, sensitivity and publication bias analyses were not carried out. Except for the quality assessment of studies, which was done with Review Manager 5.4, all data analyses were done with Stata statistical software version 17.0. Unless otherwise noted, *p* values < 0.05 were deemed statistically significant for all analyses.

## 3 Results

### 3.1 Study selection

**FIGURE 1:**
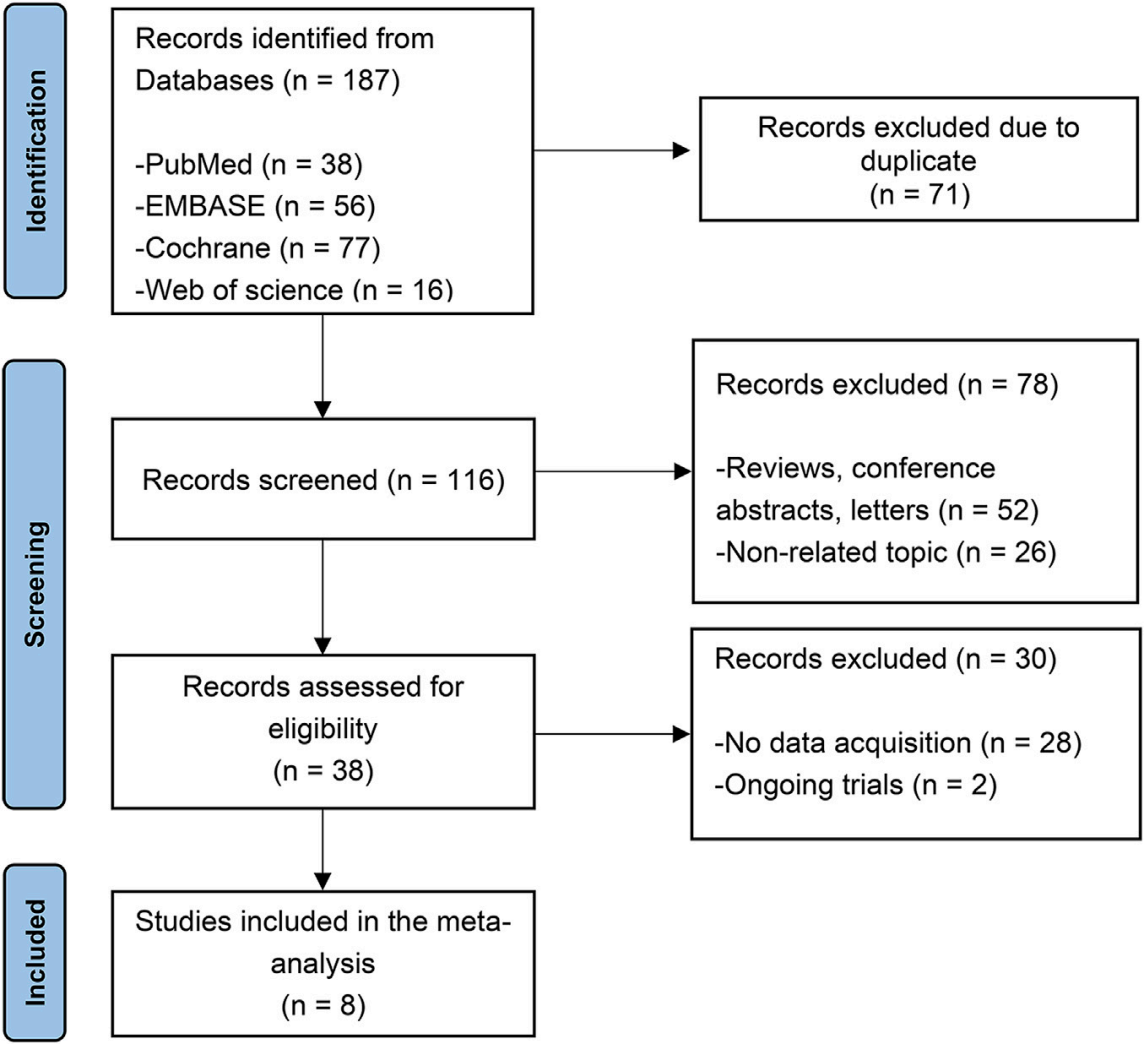
Prisma flow diagram.

The initial search turned up 187 potentially relevant studies. After removing duplicate articles, 71 papers were eliminated in total. By analyzing the titles and abstracts of the remaining 116 publications, 78 were eliminated as irrelevant. 28 were discarded due to without extractable data and two trials were still ongoing. Overall, three randomized controlled trials (RCTs) (Ferreira et al., 2016; Lees et al., 2017; Takeda et al., 2021a), three RCT extension studies (Lees et al., 2017; Ferreira et al., 2018; Takeda et al., 2021b) and three open-label studies (Reichmann et al., 2022; Santos García et al., 2022; Schofield et al., 2022) were included. The selection process is depicted in Figure 1.

### 3.2 Characteristics of included studies

Table 1 summarizes the features of the included studies. A total of 2,177 individuals with PD were involved in the study (1,404 in the long-term opicapone therapy group and 773 in the short-term opicapone therapy group). Three RCT extension studies and two open-label studies were included in the long-term group. Three RCTs and one open-label study comprised the short-term group. These studies were published between 2015 and 2022. The mean study sample size was 242, ranging between 33 and 495 participants.

**TABLE 1:** Characteristics of the included studies.

Study	Clinical study (duration)	Study design	No. of patients	Sex(male), n (%)	Age, mean ± SD, y	Dosage
Short-term						
Ferreira et al., 2015	BIPARK-I (14–15 weeks)	RCT (double-blind)	115	69 (60.0)	63.5 ± 9.2	capsule OPC 50 mg
Lees et al., 2016a	BIPARK-II (14–15 weeks)	RCT (double-blind)	150	89 (60.5)	65.5 ± 8.4	capsule OPC 50 mg
Reichmann et al., 2020	OPTIPARK (14–15 weeks)	Single arm (open-label)	363	234 (64.5)	67.8 ± 9.2	capsule OPC 50 mg
Takeda et al., 2020	COMFORT-PD (14–15 weeks)	RCT (double-blind)	145	60 (41.4)	67.4 ± 7.8	tablet OPC 50 mg
Long-term						
Schofieldet et al., 2022	OPTIPARK (24 weeks)	Single arm (open-label)	132	81 (61.4)	67.3 ± 8.4	capsule OPC 50 mg
Ferreira et al., 2018	BIPARK-I (52 weeks)	OLE (open-label)	495	-	-	capsule OPC 50 mg
García et al., 2022	OPEN-PD (24 weeks)	Single arm (open-label)	33	20(60.6)	63.3 *±* 7.9	capsule OPC 50 mg
Lees et al., 2016b	BIPARK-II (52 weeks)	OLE (open-label)	353	-	-	capsule OPC 50 mg
Takeda et al., 2021	COMFORT-PD (52 weeks)	OLE (open-label)	391	-	-	tablet OPC 50 mg

OLE, open-label extension; OPC, opicapone; RCT, randomized controlled trial.

### 3.3 Risk of bias in included studies

**FIGURE 2:**
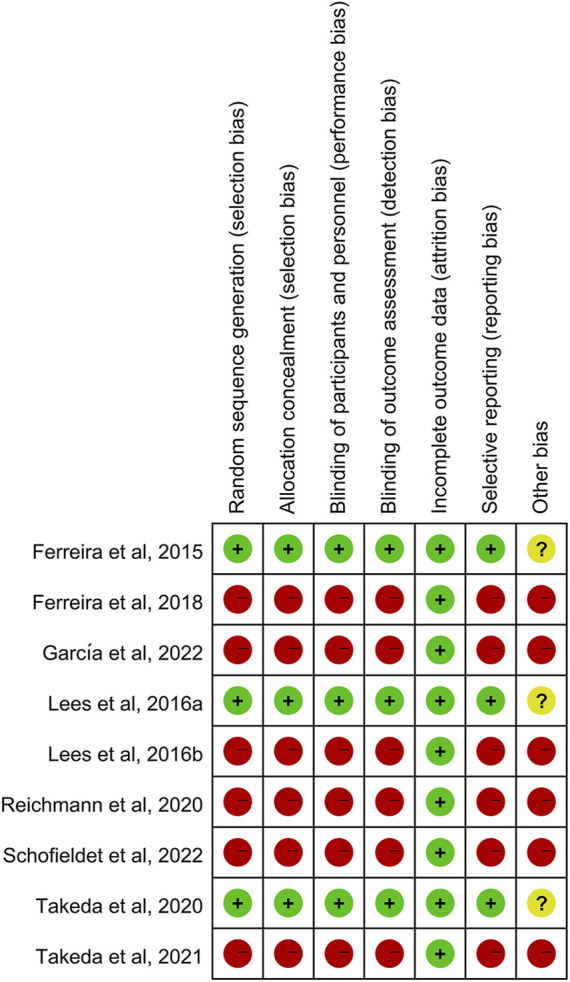
Risk of bias summary.

Risk of bias was assessed for the three RCTs, three extension studies and three open-label studies (Figure 2). Overall, the RCTs were of high quality and had a moderate risk of other bias attributed to the involvement of pharmaceutical sponsors. The quality of the extension and open-label studies was low, and there was a high risk of bias in all categories except attrition bias for all investigations.

### 3.4 Treatment-related adverse events

**FIGURE 3:**
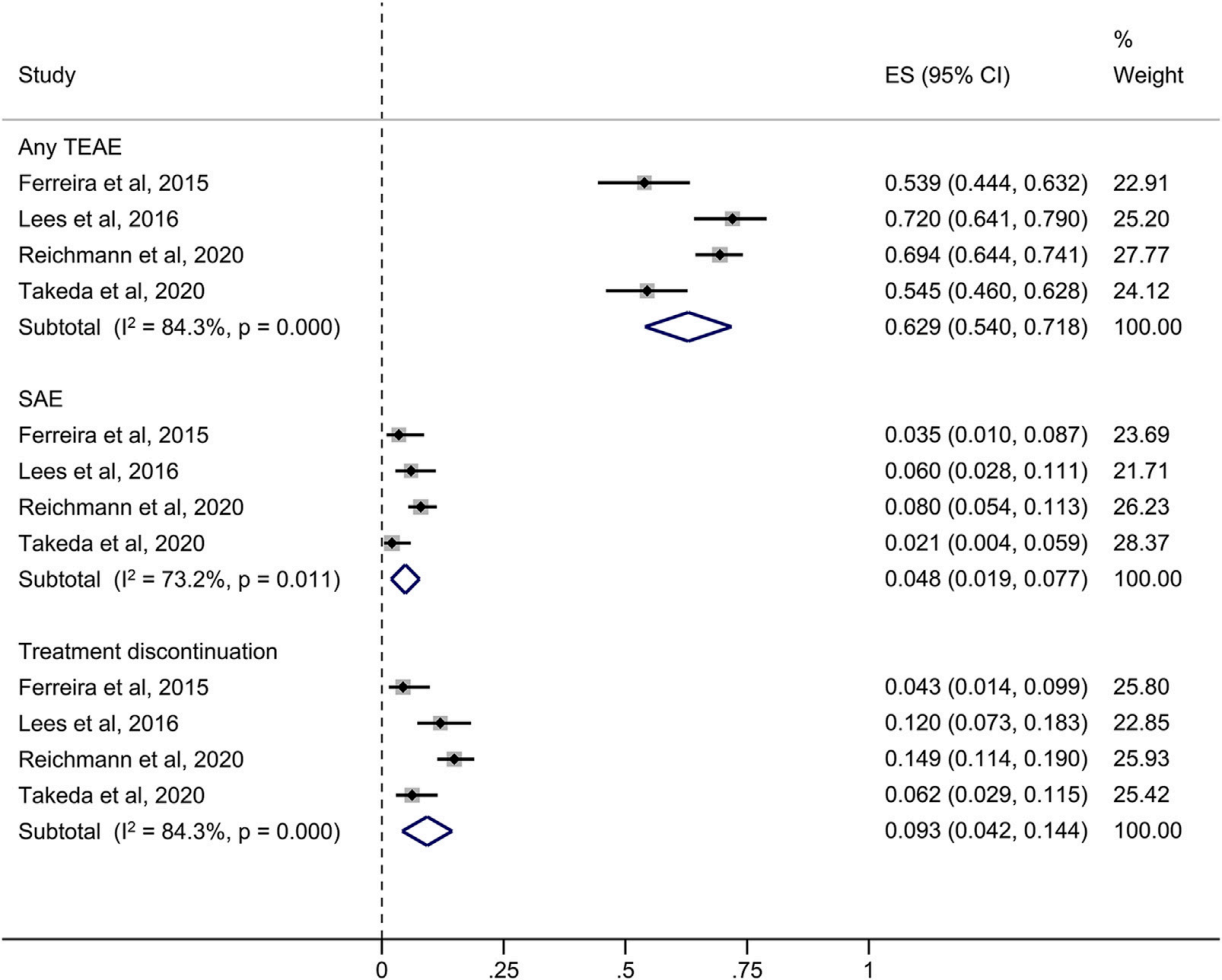
Forest plot of the pooled incidence of any TEAE, SAE and treatment discontinuation in the short-term studies. CI, confidence interval; ES, estimate; SAE, serious treatment-related adverse event; TEAE, treatment-related adverse event.

In the four short-term studies of opicapone 50 mg, 62.9% of PD patients had at least one TEAE (Figure 3). The most common TEAEs reasonably related to opicapone were dyskinesia (14.1%), elevated blood creatine phosphokinase levels (8.0%) and urinary tract infection (6.0%) as indicated in Table 2. These TEAEs were mostly classified as mild to moderate in severity.

**TABLE 2:** Treatment-related adverse events of short-term use of opicapone.

Any TEAE	Model	Studies	Event rate (%)	Lower limit	Upper limit	*Z* Value	*p* value
Dyskinesia	Random	4	14.1	0.06	0.22	3.46	0.001
CPK increased	Random	1	8.0	0.04	0.14	3.61	<0.001
Urinary tract infection	Random	1	6.0	0.03	0.11	3.09	0.002
Fall	Random	1	4.7	0.02	0.09	2.71	0.007
Constipation	Random	3	4.7	0.02	0.07	3.58	<0.001
Dry mouth	Random	2	4.3	0.03	0.06	4.79	<0.001
Dizziness	Random	2	4.2	0.02	0.06	4.61	<0.001
PD aggravated	Random	1	4.0	0.01	0.09	2.50	0.012
Hypertension	Random	1	4.0	0.01	0.09	2.50	0.012
Headache	Random	1	4.0	0.01	0.09	2.50	0.012
Nausea	Random	3	3.4	0.02	0.05	4.73	<0.001
Hallucinations	Random	2	2.5	0.01	0.04	3.51	<0.001
Insomnia	Random	2	2.0	0	0.04	2.37	0.018

CPK, blood creatine phosphokinase level; PD, Parkinson’s disease; TEAE, treatment-related adverse event.

**FIGURE 4:**
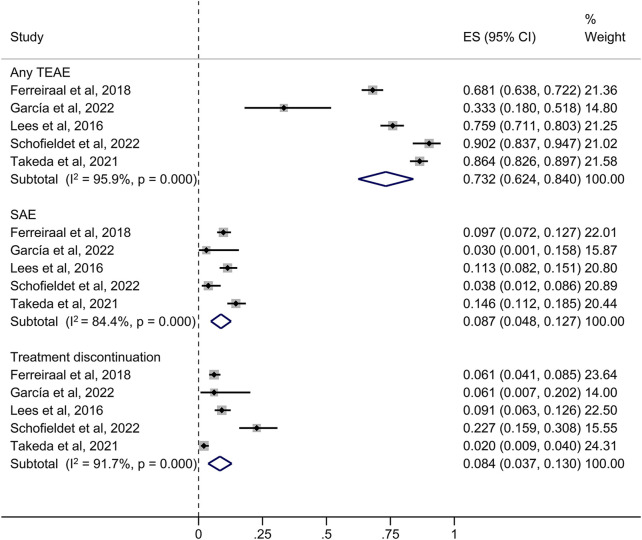
Forest plot of the pooled incidence of any TEAE, SAE and treatment discontinuation in the long-term studies. CI, confidence interval; ES, estimate; SAE, serious treatment-related adverse event; TEAE, treatment-related adverse event.

73.2% of patients in the long-term studies experienced at least one TEAE, with dyskinesia, decreased medication effect and dry mouth being the most common (Figure 4). Other TEAEs with opicapone 50 mg reported by ≥ 5% of patients included PD aggravated (7.8%), blood creatine phosphokinase level increased (7.4%), nausea (6.1%) and insomnia (5.1%). Table 3 provides an overview of the cumulative incidence of TEAEs across all trials. Remarkably, the frequencies of PD aggravation were 4% in the short-term trials and 7.8% in the extension and open-label investigations.

**TABLE 3:** Treatment-related adverse events of long-term use of opicapone.

Any TEAE	Model	Studies	Event rate (%)	Lower limit	Upper limit	*Z* Value	*p* value
Dyskinesia	Random	5	16.1	0.11	0.22	5.85	<0.001
Dry mouth	Random	1	12.1	0.07	0.19	4.27	<0.001
Drug effect decreased	Random	1	12.1	0.09	0.15	8.26	<0.001
PD aggravated	Random	3	7.8	0.00	0.16	1.96	0.050
CPK increased	Random	1	7.4	0.05	0.11	5.30	<0.001
Nausea	Random	2	6.1	0.02	0.10	3.26	0.001
Insomnia	Random	4	5.1	0.04	0.06	7.38	<0.001
Dizziness	Random	1	4.5	0.02	0.10	2.51	0.012
Abnormal dreams	Random	2	4.1	0.01	0.07	2.67	0.008
Orthostatic hypotension	Random	2	3.9	0.03	0.05	5.86	<0.001
Weight decreased	Random	1	3.8	0.02	0.06	3.95	<0.001
Constipation	Random	3	3.6	0.02	0.05	5.08	<0.001
Tremor	Random	1	3.2	0.02	0.05	4.07	<0.001
Hallucination	Random	3	3.1	0.02	0.05	4.24	<0.001
Arthritis in both wrists	Random	1	3.0	0.00	0.16	1.02	0.310
Supraspinatus tendonitis	Random	1	3.0	0.00	0.16	1.02	0.310
Unrest	Random	1	3.0	0.00	0.16	1.02	0.310
Tiredness	Random	1	3.0	0.00	0.16	1.02	0.310
OFF time increase	Random	1	3.0	0.00	0.16	1.02	0.310
Headache	Random	1	3.0	0.01	0.08	2.03	0.042
Fall	Random	2	2.2	0.01	0.03	4.18	<0.001
Contusion	Random	1	1.8	0.01	0.04	2.67	0.008
Back pain	Random	2	0.6	0.00	0.01	2.25	0.025
Nasopharyngitis	Random	2	0.5	0.00	0.01	2.20	0.028
Infuenza	Random	1	0.3	0.00	0.01	1.00	0.317
Eczema	Random	1	0.3	0.00	0.01	1.00	0.317

CPK, blood creatine phosphokinase level; PD, Parkinson’s disease; TEAE, treatment-related adverse event.

### 3.5 Serious TEAEs and treatment discontinuation

The overall rate of serious TEAEs (SAEs) in the short-term studies was 4.8%. The four studies revealed medication withdrawal due to a TEAE in 9.3% of opicapone-exposed patients with a less than 6-month follow-up, as summarized in Figure 3.

As presented in Figure 4, in the long-term (≥6 months) investigations, the incidence of SAEs was 8.7%, which included the extension and open-label studies. TEAEs resulting in premature withdrawal from studies were observed in 102 subjects (8.4%). No opicapone-related deaths were reported in both phases. The incidence of SAEs was higher in the long-term studies than in the short-term studies, although the incidence of discontinuation had the opposite pattern.

## 4 Discussion

To our knowledge, this is the first systematic review to evaluate the tolerability and safety of opicapone in the short- and long-term treatment of PD patients. Results from these eight studies in patients with PD suggest that opicapone 50 mg is well tolerated, as confirmed by relatively high completion rates in the short-term studies and low TEAE-related discontinuation rates across all studies. The majority of individuals tolerate opicapone without clinically significant effects on vital signs, cardiovascular parameters or laboratory (chemistry, hematology) assays. Patients receiving opicapone showed low rates of TEAEs in all investigations, suggesting its safety in clinic usage.

Opicapone’s extraordinarily high binding affinity reduces 3-O-methyldopa exposure, reduces maximum erythrocyte soluble COMT activity, and improves patients’ motor function (Rocha et al., 2013). Nonetheless, opicapone caused dopaminergic events by significantly boosting systemic and central levodopa bioavailability (Bonifácio et al., 2014). The most extensively researched outcome in levodopa-treated PD trials is dyskinesia. In the STRIDE-PD research on levodopa and entacapone, levodopa/carbidopa/entacapone medication had a greater frequency of dyskinesia compared to levodopa/carbidopa treatment for PD patients (41.9% vs. 31.3%) (Stocchi et al., 2010). Dyskinesia is also the most common dopaminergic event of opicapone adjuvant therapy in patients with PD. Some meta-analyses demonstrated that a brief course of opicapone medication was associated with a significantly increased incidence of dyskinesia (Kwak et al., 2022; Żegleń et al., 2022). The results of this meta-analysis showed that prolonged opicapone treatment was not associated with a higher risk of dyskinesia in PD patients.

Other dopaminergic events, such as nausea, insomnia, dizziness and hallucinations, were more prevalent among opicapone-related TEAEs in all long-term studies. These events were transient and can be alleviated by modifying the total daily dose of levodopa. Notably, long-term samples revealed higher rates of elevated blood creatine phosphokinase (7.4%), orthostatic hypotension (3.9%), arthritis in both wrists (3%) and nasopharyngitis (0.5%). Clinicians should constantly monitor changes in the patient’s condition when they are taking the medication so that appropriate action can be taken as soon as the aforementioned discomfort manifests itself. In these extension and open-label investigations, it is significant to mention that no novel or unexpected patterns in the nature, incidence or severity of TEAEs were found in individuals with PD receiving opicapone.

This study also investigated the relationship between opicapone and the risk of hepatotoxicity and gastrointestinal damage. Contrary to the first COMT inhibitor, tolcapone, which resulted in acute hepatitis and severe diarrhea (Chong et al., 2000; Borges, 2005), no evidence of an increased risk for these events was found with opicapone. In our pooled analysis, opicapone 50 mg did not appear to cause any appreciable increase in liver disorders and had no impact on the hepatobiliary laboratory indicators. In any of the two stages, there were no cases of absolutely terrible diarrhea.

## 5 Limitations

Our research has some significant limitations. The most significant difference is that there is no comparator arm with a placebo, therefore the data should be viewed with caution. Second, the minimal number of included studies and individuals in this meta-analysis could have contributed to the lack of statistical significance. Third, the average duration of follow-up was less than 1 year across all studies. Opicapone’s long-term safety is mainly unclear, which is especially important for TEAEs that can take a long time to develop, such as cancer.

## 6 Conclusion

In conclusion, opicapone appears to be an appropriate therapy for patients with PD, with good overall tolerability in both short- and long-term settings. Of note, opicapone’s favorable long-term safety and tolerability profile imply that it can be employed as a promising therapy option for patients with PD.

## Data Availability

The raw data supporting the conclusions of this article will be made available by the authors, without undue reservation.
