# Supplementary material for: Adverse event profiles of adjuvant treatment with opicapone in Parkinson’s disease: A systematic review and meta-analysis
Source: Front Pharmacol. 2022 Nov 24;13:1042992. doi: 10.3389/fphar.2022.1042992 (PMC9729693; doi:10.3389/fphar.2022.1042992)
Supplement: Supplementary file 1 [file DataSheet1.PDF]

Supplementary Material

search strategy

Supplementary Table 1. Search term and Results from Web of Science

Search #	Query	Hit: 2022-10-23
1	TS=(Parkinson Disease OR Idiopathic Parkinson's Disease OR Lewy Body Parkinson's Disease OR Parkinson's Disease, Idiopathic OR Parkinson's Disease, Lewy Body OR Parkinson Disease, Idiopathic OR Parkinson's Disease OR Idiopathic Parkinson Disease OR Lewy Body Parkinson Disease OR Primary Parkinsonism OR Parkinsonism, Primary OR Paralysis Agitans)	141896
2	TS=(Opicapone OR 2,5-dichloro-3-(5-(3,4-dihydroxy-5-nitrophenyl)-1,2,4-oxadiazol-3-yl)-4,6-dimethylpyridine 1-oxide OR ongentys OR BIA 9-1067)	425
3	TS=(Drug-Related Side Effects and Adverse Reactions OR Drug Related Side Effects and Adverse Reactions OR Drug-Related Side Effects and Adverse Reaction OR Drug Related Side Effects and Adverse Reaction OR Drug Side Effects OR Drug Side Effect OR Effects, Drug Side OR Side Effect, Drug OR Side Effects, Drug OR Adverse Drug Reaction OR Adverse Drug Reactions OR Drug Reaction, Adverse OR Drug Reactions, Adverse OR Reactions, Adverse Drug OR Adverse Drug Event OR Adverse Drug Events OR Drug Event, Adverse OR Drug Events, Adverse OR Side Effects of Drugs OR Drug Toxicity OR Toxicity, Drug OR Drug Toxicities OR Toxicities, Drug)	282155
4	#1 AND #2 AND #3	16

Supplementary Table 2. Search term and Results from Embase Database

Search #	Query	Hit: 2022-10-23
**Embase Database**	('parkinson disease'/exp OR 'lewy bodies of parkinson disease' OR 'lewy bodies of parkinson`s disease' OR 'lewy bodies of parkinsons disease' OR 'lewy body parkinson disease' OR 'lewy body parkinson`s disease' OR 'lewy body parkinsons disease' OR 'parkinson dementia complex' OR 'parkinson disease' OR 'parkinson`s disease' OR 'parkinsons disease' OR 'idiopathic parkinsonism' OR 'paralysis agitans' OR 'primary parkinsonism') AND ('opicapone'/exp OR '2, 5 dichloro 3 [5 (3, 4 dihydroxy 5 nitrophenyl) 1, 2, 4 oxadiazol 3 yl] 4, 6 dimethylpyridine n oxide' OR '5 [3 (2, 5 dichloro 4, 6 dimethyl 1 oxido 3 pyridinyl) 1, 2, 4 oxadiazol 5 yl] 3 nitro 1, 2 benzenediol' OR '5 [3 (2, 5 dichloro 4, 6 dimethyl 1 oxy pyridin 3 yl) [1, 2, 4] oxadiazol 5 yl] 3 nitrobenzene 1, 2 diol' OR 'bia 9 1067' OR 'bia 9-1067' OR 'bia 91067' OR 'bia9 1067' OR 'bia9-1067' OR 'bia91067' OR 'ongentys' OR 'opicapone') AND ('placebo'/exp OR 'placebo' OR 'placebo gel' OR 'placebos') AND ('adverse drug reaction'/exp OR 'adverse drug effect' OR 'adverse drug event' OR 'adverse drug reaction' OR 'adverse reaction, drug' OR 'drug adverse effect' OR 'drug adverse reaction' OR 'drug reaction, adverse' OR 'drug side effect' OR 'drug-related side effects and adverse reactions' OR 'long term adverse effects' OR 'metabolic side effects of drugs and substances')	56

Supplementary Table 3. Search term and Results from PubMed

Search #	Query	Hit: 2022-10-23
**PubMed**	(((（Parkinson Disease[mh]）OR（Idiopathic Parkinson's Disease）OR（Lewy Body Parkinson's Disease）OR（Parkinson's Disease, Idiopathic）OR（Parkinson's Disease, Lewy Body）OR（Parkinson Disease, Idiopathic）OR（Parkinson's Disease）OR（Idiopathic Parkinson Disease）OR（Lewy Body Parkinson Disease）OR（Primary Parkinsonism）OR（Parkinsonism, Primary）OR（Paralysis Agitans）) AND (（opicapone）OR（2,5-dichloro-3-(5-(3,4-dihydroxy-5-nitrophenyl)-1,2,4-oxadiazol-3-yl)-4,6-dimethylpyridine 1-oxide）OR（ongentys）OR（BIA 9-1067）[Title/Abstract])) AND (（Drug-Related Side Effects[Title/Abstract] AND Adverse Reactions）OR（Drug Related Side Effects[Title/Abstract] AND Adverse Reactions）OR（Drug-Related Side Effects[Title/Abstract] AND Adverse Reaction）OR（Drug Related Side Effects[Title/Abstract] AND Adverse Reaction）OR（Drug Side Effects）OR（Drug Side Effect）OR（Effects, Drug Side）OR（Side Effect, Drug）OR（Side Effects, Drug）OR（Adverse Drug Reaction）OR（Adverse Drug Reactions）OR（Drug Reaction, Adverse）OR（Drug Reactions, Adverse）OR（Reactions, Adverse Drug）OR（Adverse Drug Event）OR（Adverse Drug Events）OR（Drug Event, Adverse）OR（Drug Events, Adverse）OR（Side Effects of Drugs）OR（Drug Toxicity）OR（Toxicity, Drug）OR（Drug Toxicities）OR（Toxicities, Drug）[Title/Abstract]))	38

Supplementary Table 4. Search term and Results from Cochrane Database

Search	Query	Hit: 2022-10-23
#1	MeSH descriptor: [Parkinson Disease] explode all trees	4814
#2	(Parkinson Disease OR Idiopathic Parkinson's Disease OR Lewy Body Parkinson's Disease OR Parkinson's Disease, Idiopathic OR Parkinson's Disease, Lewy Body OR Parkinson Disease, Idiopathic OR Parkinson's Disease OR Idiopathic Parkinson Disease OR Lewy Body Parkinson Disease OR Primary Parkinsonism OR Parkinsonism, Primary OR Paralysis Agitans):ti,ab,kw (Word variations have been searched)	11961
#3	#1 or #2	11961
#4	(Opicapone):ti,ab,kw	261
#5	(Opicapone OR 2,5*dichloro*3*(5*(3,4*dihydroxy*5*nitrophenyl)*1,2,4*oxadiazol*3*yl)*4,6*dimethylpyridine 1*oxide OR ongentys OR BIA 9*1067):ti,ab,kw (Word variations have been searched)	264
#6	#5 or #4	264
#7	MeSH descriptor: [Drug-Related Side Effects and Adverse Reactions] explode all trees	3895
#8	(Drug-Related Side Effects and Adverse Reactions OR Drug Related Side Effects and Adverse Reactions OR Drug-Related Side Effects and Adverse Reaction OR Drug Related Side Effects and Adverse Reaction OR Drug Side Effects OR Drug Side Effect OR Effects, Drug Side OR Side Effect, Drug OR Side Effects, Drug OR Adverse Drug Reaction OR Adverse Drug Reactions OR Drug Reaction, Adverse OR Drug Reactions, Adverse OR Reactions, Adverse Drug OR Adverse Drug Event OR Adverse Drug Events OR Drug Event, Adverse OR Drug Events, Adverse OR Side Effects of Drugs OR Drug Toxicity OR Toxicity, Drug OR Drug Toxicities OR Toxicities, Drug):ti,ab,kw (Word variations have been searched)	228312
#9	#7 or #8	229289
#10	#3 and #6 and #9 in Trials	77
